# Structural properties and thermal stability of cobalt- and chromium-doped α-MnO_2_ nanorods

**DOI:** 10.3762/bjnano.8.104

**Published:** 2017-05-10

**Authors:** Romana Cerc Korošec, Polona Umek, Alexandre Gloter, Jana Padežnik Gomilšek, Peter Bukovec

**Affiliations:** 1 Faculty of Chemistry and Chemical Technology, University of Ljubljana, Večna pot 113, SI-1000 Ljubljana, Slovenia; 2 Jožef Stefan Institute, Jamova cesta 39, SI-1000 Ljubljana, Slovenia; 3 Laboratoire de Physique des Solides, Université Paris Sud, CNRS UMR 8502, F-91405 Orsay, France; 4 Faculty of Mechanical Engineering, University of Maribor, Smetanova 17, SI-2000 Maribor, Slovenia

**Keywords:** α-MnO_2_, doping, EXAFS, nanorods, XANES

## Abstract

α-MnO_2_ nanorods were synthesized via the hydrothermal decomposition of KMnO_4_ in an acidic environment in the presence of Co^2+^ and Cr^3+^ ions. Reactions were carried out at three different temperatures: 90, 130 and 170 °C. All prepared samples exhibit a tetragonal MnO_2_ crystalline phase. SEM–EDS analysis shows that cobalt cations are incorporated to a higher degree into the MnO_2_ framework than chromium ions, and that the content of the dopant ions decreases with increasing reaction temperature. The oxidation of Co^2+^ to Co^3+^ during the reaction was proved by an XANES study, while EXAFS results confirm that both dopant ions substitute Mn^4+^ in the center of an octahedron. The K/Mn ratio in the doped samples synthesized at 170 °C is significantly lower than in the undoped samples. Analysis of an individual cobalt-doped α-MnO_2_ nanorod with HAADF-STEM reveals that the distribution of cobalt through the cross-section of the nanorod is uniform. The course of thermal decomposition of the doped nanorods is similar to that of the undoped ones. Dopant ions do not preserve the MnO_2_ phase at higher temperatures nor do they destabilize the cryptomelane structure.

## Introduction

The wide range of physical and chemical properties of manganese dioxide (MnO_2_), which exists in several polymorphic forms, originates from the different structures in which MnO_6_ octahedrons are linked by edge- or corner-sharing in different ways to form layered or channel structures [1–2]. The negative charge of the Mn–O network arises from the mixed oxidation states of manganese (Mn^4+^, Mn^3+^, and Mn^2+^). It is compensated with hydrated cations that are incorporated inside the pores in the case of tunnel structures, while they are situated between the layers in the layered structures. The tunnel cations are exchangeable, meaning that these structures can serve as ion or molecular sieves [3]. The size and shape of different tunnel sizes (1 × 1, 1 × 2, 2 × 2, 3 × 3, 3 × 2, 2 × 4) direct the pore opening and control the separation [1,4]. In α-MnO_2_, octahedrons form the 2 × 2 channel structure with a pore size of 4.6 Å [5]. In the structure named cryptomelane hydrated K^+^ cations are responsible for the electroneutrality, while in hollandite the tunnels are occupied with Ba^2+^ cations.

Cryptomelane MnO_2_ with the chemical composition KMn_7_^4+^Mn^3+^O_16_·*n*H_2_O is the most extensively studied octahedral manganese oxide molecular sieve. A minor amount of Mn^3+^ replaces Mn^4+^ in the center of the octahedrons, leading to an average oxidation state of 3.9, which is common for 2 × 2 tunnel structures [6–7]. The numerous studies are based on the fact that this low-cost and environmentally friendly material possesses some excellent properties: conductivity, microporosity, and catalytic activity [1]. It can be used as a cathode-active material for rechargeable lithium batteries [8], an electrode material for supercapacitors [9–10], and shows excellent catalytic activity for the selective oxidation of benzyl alcohols [11–12]. The catalytic properties are related to the redox cycling of various oxidation states of manganese [1], while the partial exchange of K^+^ with protons yielded excellent acid catalysts for selective oxidation reactions [13]. The incorporation of metal cations with different valencies (Cu^2+^, Co^2+^, Ni^2+^, Ag^+^, V^5+^, W^6+^, Mo^6+^) into the channels or in the cryptomelane structure produces materials with novel morphologies and enhanced catalytic properties [14–15].

The 2 × 2 tunnel structures, prepared by different methods, showed different thermal properties that depend on the average oxidation state of manganese, the type and content of cations situated in the tunnels, and also on the number of defects causing structural/lattice constraints [5,16–17]. The structure without any large stabilizing cations is thermally stable up to about 480 °C. Upon further increase of the temperature, the tunnel framework collapses and transforms into Mn_2_O_3_ with a dense bixbyite structure [18]. In cryptomelane, the phase transformation MnO_2_→Mn_2_O_3_ is limited to a maximal temperature of 800–900 °C, indicating that K^+^ ions enhance the structural stability [17]. This is of great importance in some applications where local overheating could cause structural changes. In some cases, doped cryptomelane samples exhibit a higher thermal stability than undoped ones. For instance, doping with tin and cobalt shifted the reduction of α-MnO_2_ to Mn_2_O_3_ from 500–550 °C (undoped sample) to 850–900 °C [19–20]. This was ascribed to the incorporation of dopant ions into tunnels stabilizing the structure [19]. Doping with Al^3+^ and Mg^2+^ also slightly increases the temperature of the thermal transformation of MnO_2_ to Mn_2_O_3_ (from 500 °C for undoped samples to 590 °C for doped ones), while chemical analysis showed an increased content of K^+^ ions in these samples. In contrast, doping with Cu^2+^ lowered the content of K^+^ ions and also decreased thermal stability [8]. Undoped materials also possess a higher thermal stability than materials doped with one or more elements of Fe^3+^, Cu^2+^, Mo^6+^, V^5+^ with the exception of that doped only with Fe^3+^, which shows the same stability as the undoped samples [14,21]. The introduction of silver ions into the cryptomelane structure also lowered thermal stability due to a partial distortion of the regular channel-like structure [22].

The influence of doping the pristine material with different ions on its thermal stability is quite complex. When an ion of higher valence (3+, 2+, 1+) substitutes Mn^4+^ or Mn^3+^ in the cryptomelane structure, several different structural changes can take place: (I) Due to the more negative charge of the framework more K^+^ ions are incorporated into tunnels, leading to enhanced thermal stability. The dopant ions should be of very similar size, causing no structural distortion. The latter would lead to lower symmetry, from tetragonal to monoclinic, which means also a lower thermal stability. (II) Dopant ions of lower valence lead to the formation of octahedral vacancies to maintain the charge balance, with or without the additional incorporation of K^+^ into tunnels. Vacancies cause structural distortion and lower the thermal stability. When ions of higher valence are incorporated, the content of K^+^ ions is reduced or more Mn^4+^ ions are transformed to Mn^3+^ [14].

Recently, a few different strategies of doping of different MnO_2_ structures with Co^2+^ have been published. It seems that doping with cobalt allows for the preparation of materials with enhanced characteristics. In an attempt to modify α-MnO_2_ as cathode for high energy density lithium batteries, nanostructured MnO_2_, doped with 2 atom % Co, was synthesized [20]. In Co-doped birnessite-type MnO_2_ nanoparticles, synthesized under ambient conditions from KMnO_4_ and ethylene glycol, doping with Co prevented agglomeration and increased the specific surface area. The prepared materials possess a very high specific capacity and are potential candidates for supercapacitors [23]. Co-doped ramsdelitte MnO_2_ with 1 × 2 tunnel structure could be also used for this purpose. Nanoflakes of this material, arranged in the yolk–shell secondary structure, can be prepared through a simple one-pot synthesis of the precursor solution irradiated with UV light. The incorporation of cobalt into the structure improved the electrical conductivity, while nanoflakes and the secondary structure increase the specific surface area, leading to improved electrode kinetics by facilitating mass transport [24]. However, there is a lack of detailed structural studies of these materials in order to understand why cobalt as dopant so drastically affects the physical properties of the MnO_2_ matrix.

Because of economic reasons MnO_2_ is one of the most attractive cathodes for rechargable lithium batteries. Since Mn is the tenth-most abundant element in the Earth crust, lithiated MnO_2_ cathodes would cost around 1% of LiCoO_2_, the cathode material of choice in rechargable Li-ion batteries. Under overcharge conditions Mn^4+^ is much more safer than Co^4+^ and Ni^4+^, which are thermally unstable at the top charge. Also, at higher energy densities and operation voltages, and in combination with organic flammable electrolytes and carbon anodes in a cell, MnO_2_ increases safety margins [8].

In this work, comprehensive studies about the structural and thermal stability of α-MnO_2_ doped with Co^3+^ and Cr^3+^ were carried out and compared with results of undoped samples. The mentioned dopant ions were chosen because they have a similar ionic radius as Mn^4+^and therefore are expected to readily replace Mn^4+^ in MnO_2_ matrix. The replacement of Mn^4+^ with dopant ions (Co^2+^, Cr^3+^) would consequently increase the negative charge of the network and the concentration of K^+^ in the tunnels, which in turn would lead to an improved thermal stability. This is especially important in some of the previously mentioned technological applications. The samples were prepared by hydrothermal synthesis at three different temperatures. Dopant content as well as the content of manganese and potassium were determined using scanning electron microscopy, coupled with an energy dispersive X-ray spectrometer (SEM–EDS). Obtained results were correlated with XAFS and XANES (X-ray absorption fine structure; X-ray absorption near-edge structure) analysis, which helps to determine the position of dopant ions in the cryptomelane structure, as well as the oxidation states of manganese and dopant ions. These parameters proved to be crucial for understanding the structure of the synthesized nanorods and their thermal stability. Gasses evolved during thermogravimetric analysis (TG) were detected by coupling a TG instrument with a mass spectrometer. The influence of reaction temperature on the secondary structure, morphology, and crystal structure of the obtained products was also studied.

## Results and Discussion

**Figure 1:**
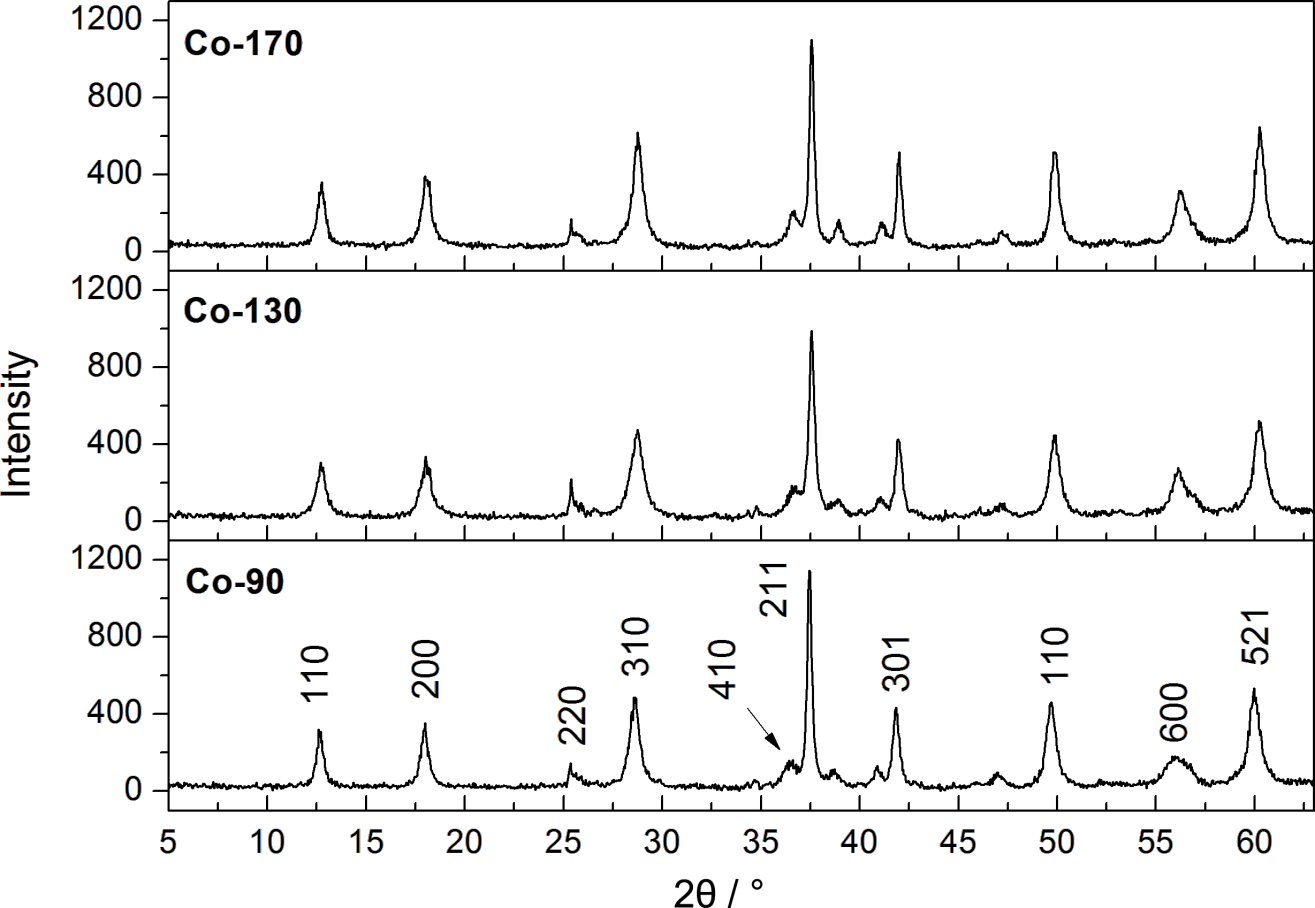
X-ray diffractograms of the samples doped with cobalt synthesized at 90 °C (**Co-90**), 130 °C (**Co-130**) and 170 °C (**Co-170**).

The phase identification of the synthesized samples was performed using powder X-ray diffraction. All diffraction patterns correspond to the tetragonal phase of α-MnO_2_ (space group *I*4/*m*; JPCDS 44-0141) and, in the case of the doped samples, show no additional peaks of crystalline phases belonging to the cobalt or chromium oxides (Figure 1). In order to evaluate the impact of the reaction temperature on the unit cell size of α-MnO_2_, the unit cell parameters *a* and *c* were extracted from the XRD patterns (Table 1). Interestingly, the lattice parameter *a* systematically decreases with increasing reaction temperature for all three reaction batches (undoped, cobalt- and chromium-doped) while the lattice parameter *c* does not show any dependence on the reaction temperature. In addition, a significant difference was observed in the lattice parameter *a* of the cobalt-doped samples synthesized at 90 °C. The XANES results (see below) indicate lower oxidation states of both manganese and cobalt in this sample. This is most probably the reason for the larger lattice parameter *a*.

**Table 1:** Reaction conditions, chemical composition, and structural parameters for the undoped and doped α-MnO_2_ samples.

sample	reaction temperature (°C)	elemental composition (atom %)						lattice constants (Å)	
		K	Mn	O	S	Co	Cr	*a*	*c*

**ND-90**	90	2.6	26.7	69,3	1.4	—	—	9.823	2.852
**ND-130**	130	3.5	26.5	68.9	1.1	—	—	9.808	2.853
**ND-170**	170	3.8	26.2	68.7	1.3	—	—	9.802	2.855

**Co-90**	90	2.4	24.7	69.6	2.0	1.3	—	9.850	2.856
**Co-130**	130	3.5	26.0	68.3	1.4	0.8	—	9.809	2.849
**Co-170**	170	3.5	25.0	68.4	2.3	0.6	—	9.799	2.851

**Cr-90**	90	2.8	28.8	66.4	1.7	—	0.3	9.826	2.852
**Cr-130**	130	3.8	28.7	65.4	1.9	—	0.2	9.812	2.853
**Cr-170**	170	3.0	24.4	69.4	3.1	—	0.1	9.803	2.854

**Figure 2:**
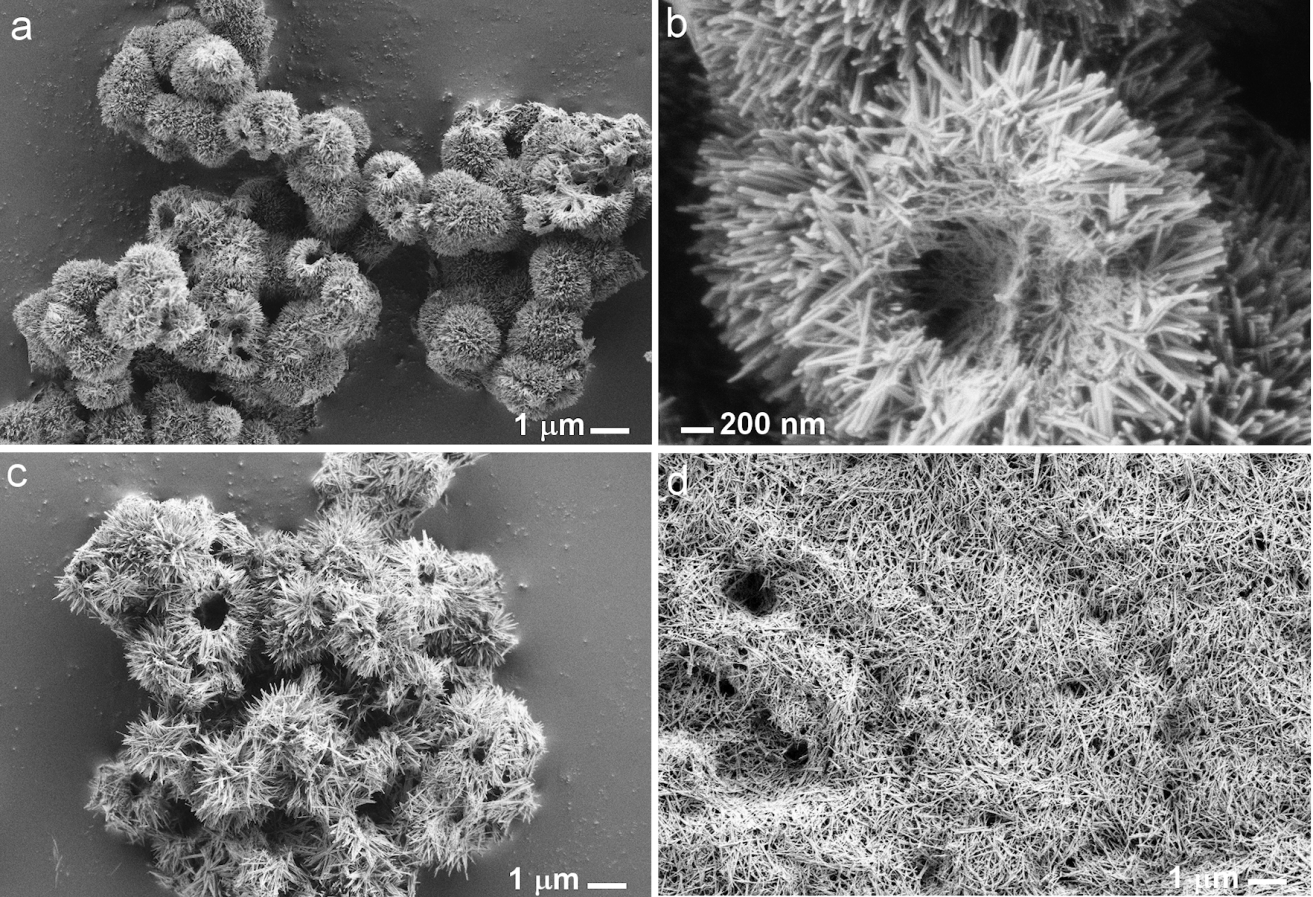
FE-SEM images of the cobalt-doped α-MnO_2_ samples synthesized at 90 °C (a), 130 °C (c) and 170 °C (d), and image of a hollow microstructure of the **Co-90** sample (b). Images a, c, and d were taken at the same magnification.

Next, morphological studies of the samples were conducted by field emission scanning microscopy (FE-SEM) and transmission electron microcopy (TEM). As revealed in the SEM images, **Co-90** and **Co-130** possess two degrees of hierarchy: (i) nanorods as a primary structure that (ii) form hollow microstructures at a the secondary level (Figure 2a,c) similar to Fe^3+^-doped α-MnO_2_ nanotubes [25]. Characteristic diameters of these microstructures are between 2 to 5 μm. The secondary structure appears to be more disordered already at 130 °C (Figure 2c), while it is not observed at all at 170 °C (Figure 2d). The same behavior was seen in the series of the chromium-doped and the undoped α-MnO_2_ samples.

A more detailed inspection of the microstructure shells of the **Co-90** sample (Figure 2b) shows that they are constructed from thicker and thinner nanorods. Thicker nanorods are found on the outer side of the shells while thinner ones are in the inner part. In general, the length of the nanorods corresponds to the shell thickness of the hollow microstructures, which is between 400 nm and 1 μm. In contrast, nanorods in the samples synthesized at 130 and 170 °C can reach up to 2 μm, which is in agreement with our findings reported in the study of the impact of the reaction conditions on dimensions of α-MnO_2_ nanorods [26]. In addition, all samples appear to be very homogenous regarding morphology.

**Figure 3:**
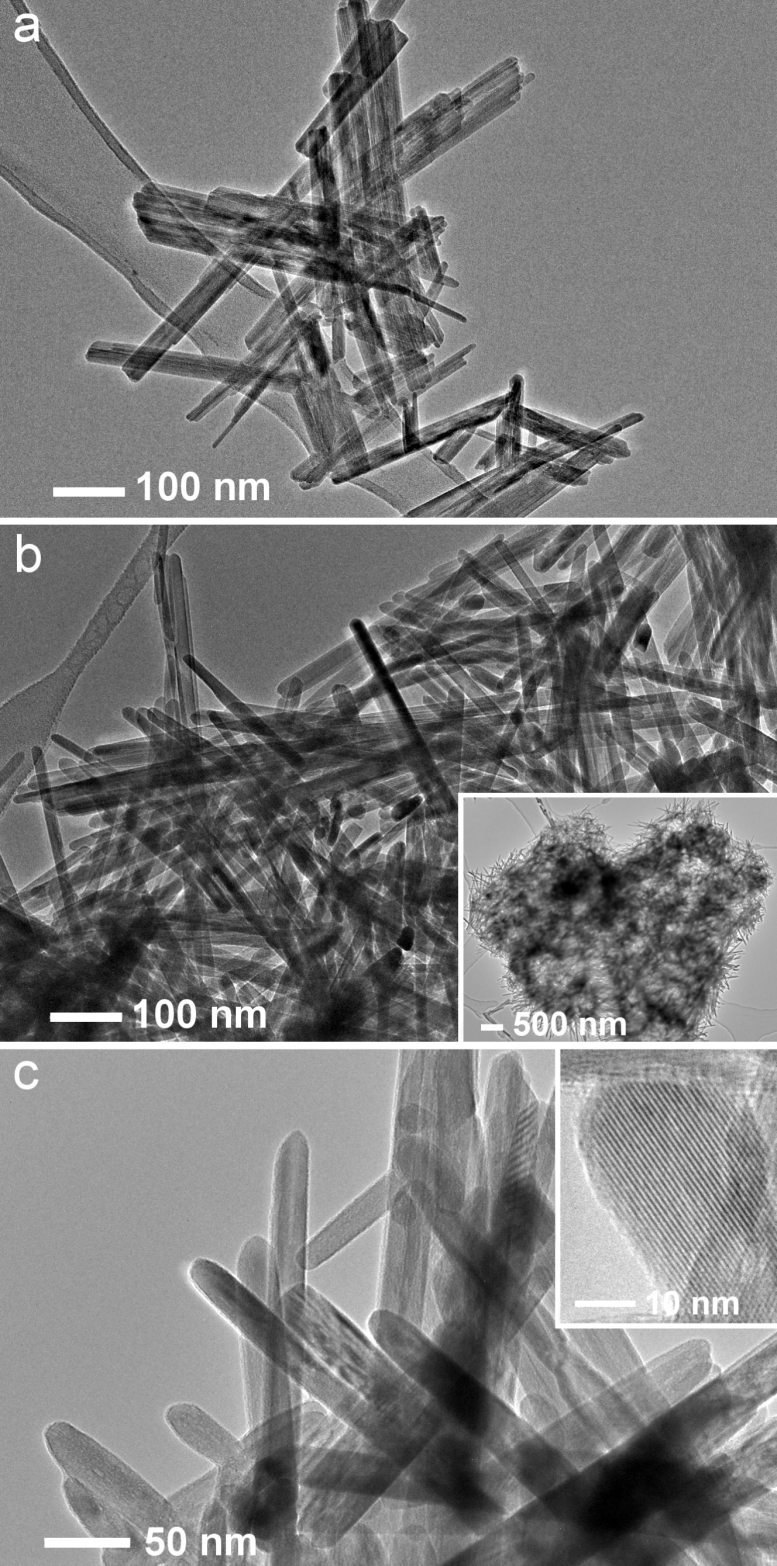
TEM images of chromium-doped α-MnO_2_ nanorods synthesized at 90 °C (a), 130 °C (b), and 170 °C (c).

Information on the diameter and crystallinity of the synthesized nanorods was obtained from analysis of TEM images (Figure 3). Diameters of the thinner nanorods are in the range from 9 to 15 nm, whereas the diameter of the thicker ones is between 20 and 35 nm. The thinner nanorods were not observed in the samples synthesized at 170 °C. Nanorods in all synthesized samples are crystalline. Clear lattice fringes of 0.68 nm are observed (Figure 3c) corresponding to the interplanar spacing of (110) planes in α-MnO_2_ (JPCDS 44-0141).

**Figure 4:**
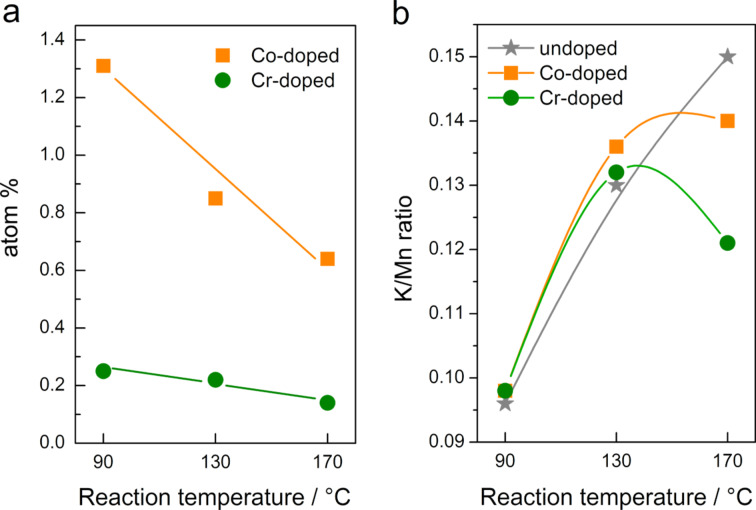
Dependence of chromium and cobalt content (a) and K/Mn (atom %) ratio (b) on the reaction temperature.

The elemental composition of the samples was carried out by energy dispersive X-ray spectroscopy (EDS), and the results are shown in Figure 4. Interestingly, much more cobalt than chromium is incorporated into the α-MnO_2_ matrix although the initial molar concentration of both ions in the reaction mixture was the same. In fact, the chromium content in all three samples is below 0.3 atom %. The low chromium content also explains why the unit cell parameter *a* of these samples does not differ significantly from the values of the undoped samples (Table 1). Moreover, the content of both ions decreases with increasing reaction temperature, meaning that at higher reaction temperatures the ability of the dopant ions to incorporate into an α-MnO_2_ matrix is reduced (Figure 4a). The measured spectra show no signs of radiation damage of the samples: subsequent spectra measured at the same spot are, except for the noise, identical to the first one. No change in the oxidation state or in the structure is detected. The summed spectra are used for further analysis.

**Figure 5:**
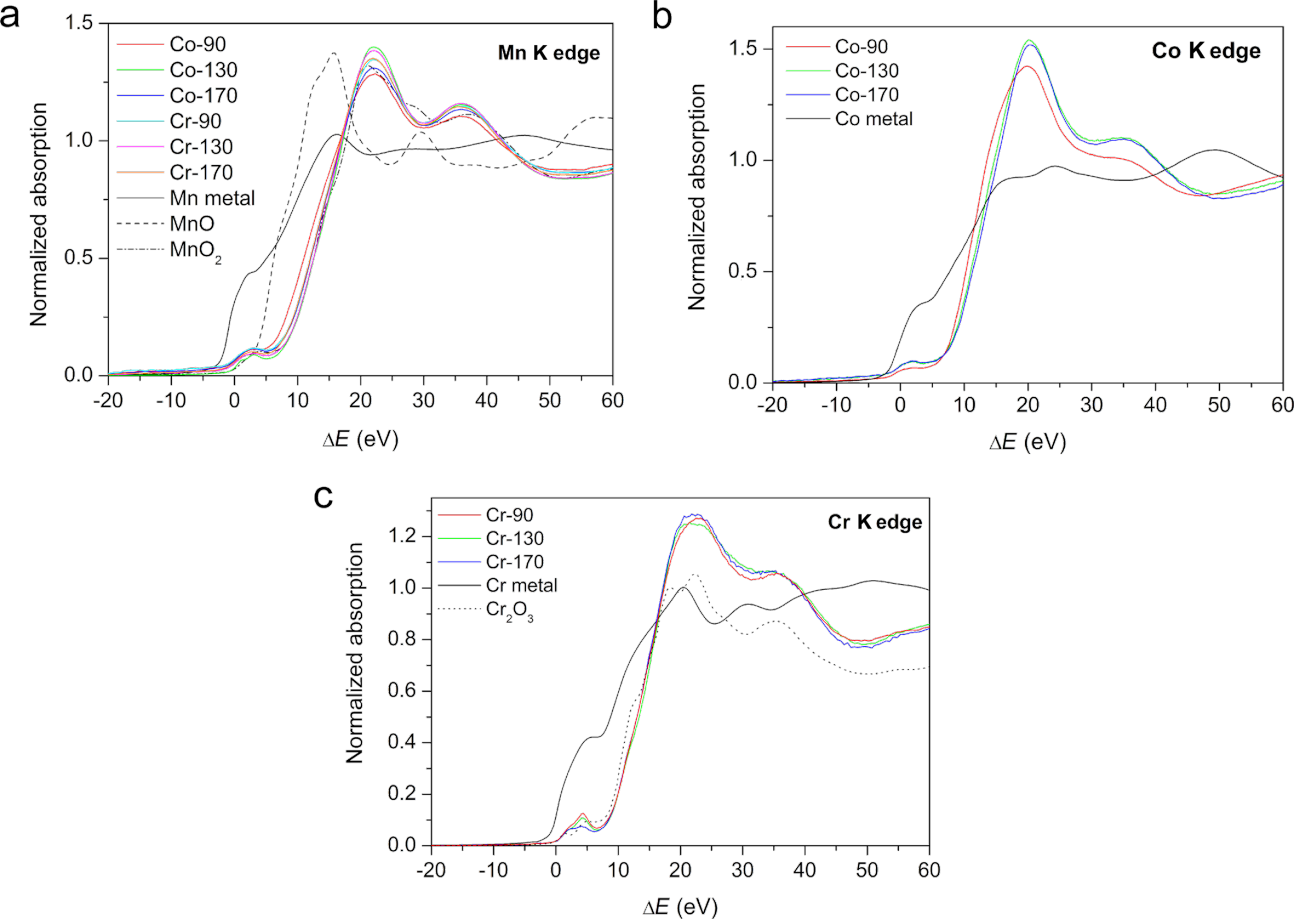
XANES data at the Mn edge (a), the Co edge (b) and the Cr edge (c) for all synthesized samples together with spectra of Co and Cr metals and corresponding oxides (MnO, MnO_2,_ and Cr_2_O_3_).

Figure 5 shows X-ray absorption near edge structure (XANES) data: K-edge profiles of Mn, Co and Cr for all samples together with the spectrum of the corresponding metal and some of its oxides. Clearly, all Mn sample spectra agree with the MnO_2_ spectrum [27], the oxidation state of Mn is 4+. Although there was definitely around 10% of Mn^3+^ present in the MnO_6_ framework [6–7], the presence of the reduced species was not confirmed because of the detection limit of the XANES measurement, which was of the same order. The oxidation state of Co^3+^ can be deduced from a comparison with CoO and Co_2_O_3_ spectra [28], and Cr is 3+ as in Cr_2_O_3_ [29]. The spectra of the samples at the same edge are very similar, showing no major changes of metal coordination during the heating. There is a slight shift of the Mn and Co edges in the **Co-90** sample toward lower energies, i.e., to a smaller oxidation number. In the spectrum of **Cr-170**, the Cr K edge pre-peak at Δ*E* ~ 4 eV is of slightly different shape than in the other two spectra, indicating possible distortion of the symmetry of the Cr site.

**Figure 6:**
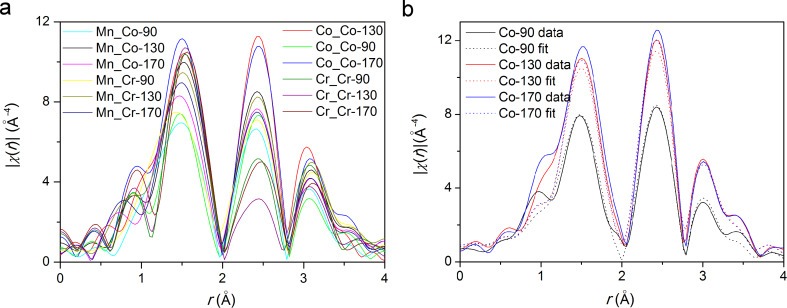
EXAFS spectra of all samples, *k* = 4–11 Å^−1^, *k*^3^ weighing, in *r* space (a) and spectra at Co edge with their best fit models, combined *k*^1^ + *k*^2^ + *k*^3^ weighing, *k* = 3.5–11.5 Å^−1^, *r* = 1.2–3.5 Å (b).

All EXAFS spectra are very similar, showing three distinct peaks in the *r* region up to 4 Å (Figure 6a), suggesting that Co and Cr dopant ions replace Mn ions in the structure, i.e., each of the three metal ions is surrounded by the same configuration of neighbors. However, the heights of the peaks and their exact positions differ.

Using XRD results, we built an EXAFS model of α-MnO_2_ [30]. In Co and Cr K-edge models, the central Mn ion was replaced with the corresponding dopant ion. Due to the low dopant concentration (Figure 4a) all neighboring metal ions are expected to be manganese, the contribution of dopant neighbors is below the detection limit of the analysis. In the first modelling cycle, each model comprised the strongest single scattering paths of α-MnO_2_: 4 O paths of the six oxygen ions at 2.88–2.92 Å, 2 Mn paths of 4 Mn ions at 2.87–2.91 Å and one Mn path with 4 Mn ions at 3.43 Å. In the evolved models, we combined the paths into three groups, corresponding to three peaks in the *r*-space (Figure 6a): the first shell of oxygen neighbors and two shells of manganese neighbors. Comparison of the best fit parameters showed negligible destructive interference of the paths in the individual group and therefore no loss of essential information.

The parameters obtained in the separate fits were similar enough to suggest simultaneous fits of all spectra of the same ion. In the three final models, the number of oxygen neighbors was set to 6 in all cases. For all spectra in the model, we used the same values for the energy shift *E*_0_, the amplitude parameter *S*_0_^2^, path lengths *r_i_* and coordination numbers *N_i_* of manganese neighbors, while the Debye–Waller factors σ_0_^2^ were left as free parameters. Good fits were obtained, confirming the consistency of the models. Figure 6b shows a typical fit data at one of the edges (Co).

The applied model describes the differences between the samples mostly as differences in the degree or structural order. In this view , the **Co-90** sample – with the smallest amplitude of the first peak in the Fourier transform (at ca. 1.5 Å^−1^ in Figure 6a), therefore requiring the highest Debye–Waller factor (not shown) to agree with the common *S*_0_^2^ factor of the modelled group (Table 2) – shows the most distorted O shell around both metal ions and also the most distorted Mn shells around the Co ion. The **Co-90** sample also differs from the other two Co samples in XRD (Table 1) and XANES spectra (Figure 5b). Due to the high correlation between *N* and σ_0_^2^, some reduction in the number of O neighbours cannot be excluded.

**Table 2:** Best fit parameters obtained by modelling the EXAFS spectra. The uncertainty intervals in units of the last decimal place are given in parentheses. Values without error estimate are held fixed or constrained in the relaxation.

	Cr^3+^	Mn^4+^	Co^3+^

*S*_0_^2^	0.63(8)	0.68(3)	0.72(9)

*N*_1_ (O)	6	6	6
*r*_1_ [Å]	1.96(1)	1.89(1)	1.91(1)

*N*_2_ (Mn)	2.7(20)	5.5(4)	4.3(14)
*r*_2_ [Å]	2.91(1)	2.88(1)	2.84(1)

*N*_3_ (Mn)	2.5(19)	2.1(3)	2.0(16)
*r*_3_ [Å]	3.46(1)	3.44(1)	3.42(1)

The Co–O and Cr–O distances found in the best fit EXAFS models (Table 2) are larger than the Mn–O distance by 0.02 Å and 0.07 Å, respectively. This is in total agreement with the values of 6-coordinate octahedral ionic radii of Mn^4+^, Co^3+^ and Cr^3+^ ions [31], confirming the XANES findings of the metal oxidation states.

As already mentioned, doping of the α-MnO_2_ structure can take place either in the Mn–O framework where dopant ions substitute manganese ions, or in the tunnels where they substitute potassium cations. This depends on the ionic radii of the dopant ions and their coordination [14,32]. The size of sixfold-coordinated Co^3+^ (0.615 Å) and Cr^3+^ (0.755 Å) is very close to the size of the sixfold-coordinated Mn^4+^ (0.67 Å). Therefore, for Co^3+^ and Cr^3+^, it is more likely that the substitution of manganese atoms in the Mn–O framework will take place than the substitution of potassium (1.65 Å) located in the tunnels. As already shown, the EXAFS analysis confirmed this assumption. The reason that Co^3+^ ions are incorporated to a higher degree into the Mn–O framework than Cr^3+^ ions is possibly the smaller size of the former.

In Figure 4b, the K/Mn ratio as a function of the reaction temperature is shown for all synthesized samples. It is immediately apparent that this ratio increases with increasing reaction temperature for the undoped samples. This may indicate that the amount of manganese atoms in oxidation states lower than 4+ is increasing. The K/Mn ratio of the doped samples is similar to the undoped samples for the samples synthesized at 90 and 130 °C while a significant difference is observed for the samples synthesized at 170 C. The discrepancy is largest for **Cr-170**. Since the doping level of cobalt and chromium in **Co-170** and **Cr-170** is the lowest (Figure 4a). This might be an indication that at 170 °C the dopant ions partially substitute K^+^ in the tunnels. The EXAFS spectrum of this sample also indicates a distortion of the symmetry of the chromium site. In addition, all samples contain sulfur. The average sulfur content falls in the range between 1.1 and 3.1 atom % (Table 1). The sulfur detected in the samples is originating from the sulfuric acid that was a part of the reaction mixture [26].

**Figure 7:**
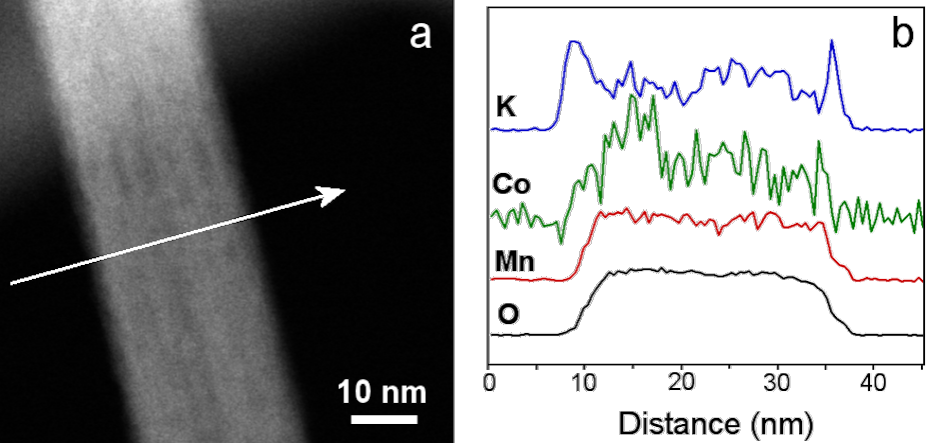
HAADF-STEM image of a cobalt-doped MnO_2_ nanorod synthesized at 90 °C (a) and chemical profile obtained from EELS analysis of the K K, Co L, Mn L and O K edges (b) along the arrow shown in panel a.

The chemical composition of an individual nanorod (**Co-90**) was determined using electron energy loss spectroscopy (EELS) in combination with high-angle annular dark field scanning transmission electron microscopy (HAADF-STEM), both shown in Figure 7. The chemical profiles of manganese and oxygen show a correlation with the nanorod shape while the cobalt profile shows more fluctuations due to the rather lower cobalt content (1.3 atom %). Nevertheless, it is clear that cobalt is present throughout the cross section of the nanorod. However, the chemical profile of potassium shows higher potassium content at the nanorod edges, indicating that more K^+^ ions are adsorbed at the surface of the nanorod. A similar analysis of the chromium-doped samples could not be performed because of the small content of chromium ions (below 0.3 atom %), which is below the detection limit.

**Figure 8:**
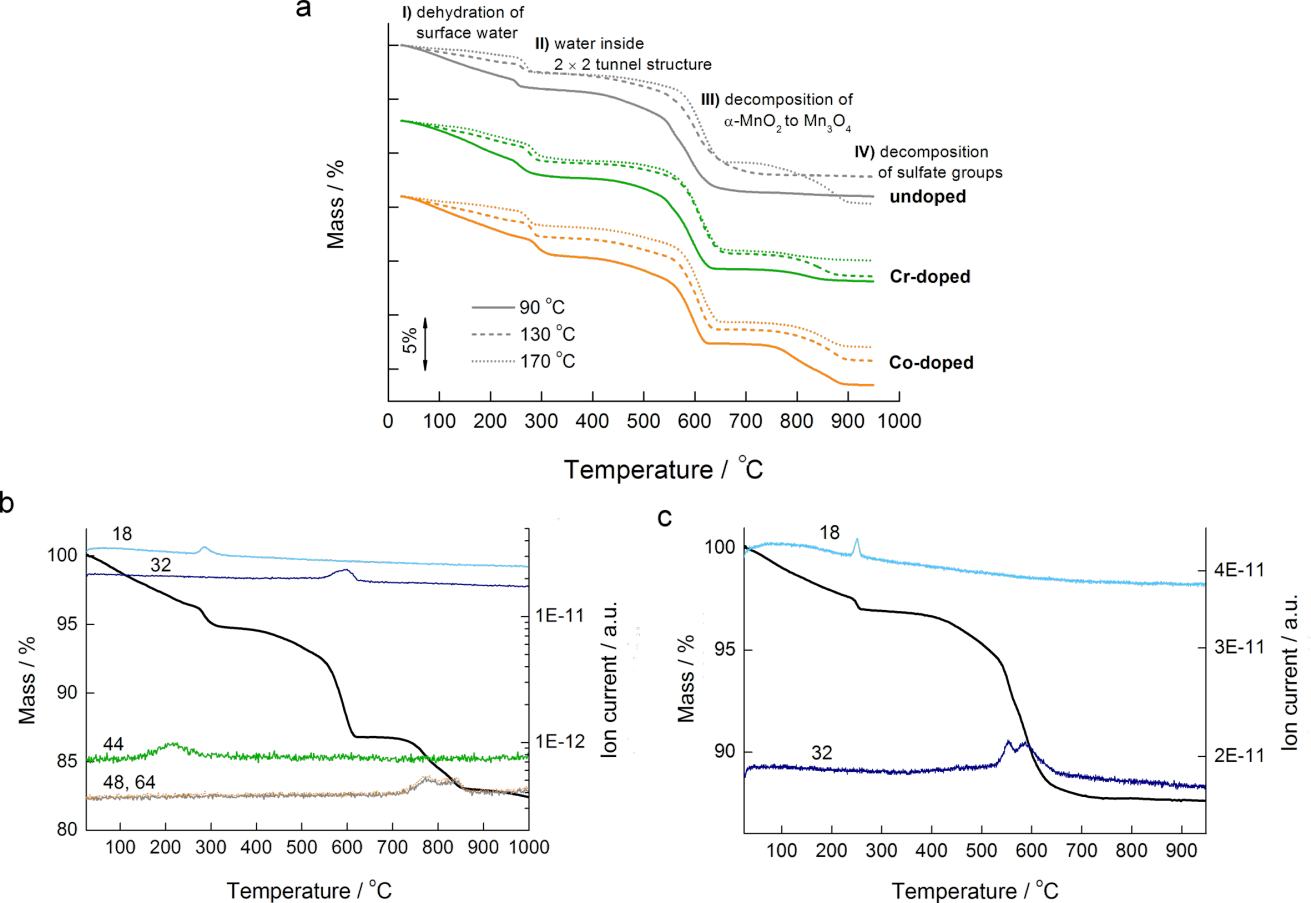
Dynamic TG curves in an inert atmosphere of undoped, chromium- and cobalt-doped samples synthesized at 90, 130 and 170 °C, respectively (a); TG-MS curve of **Co-90** (b) and TG-MS curve of **ND-90** (c).

Comparison of TG curves (Figure 8a) shows that the course of thermal decomposition of chromium- and cobalt-doped samples is similar to that of the undoped samples. In our case, the dopant ions did not preserve the MnO_2_ phase at higher temperatures. This behavior was expected due to the low content of incorporated dopant ions. The main difference between the samples is in the content of adsorbed water, which is released between room temperature and around 250 °C together with the solvent (methanol). The water content varies from 1.42% (**ND-170**) to 3.95% (**Cr-90**). More water molecules are released from the samples prepared at the lowest reaction temperature (90 °C). In these samples, nanorods form hollow 3D microstructures that can also keep water molecules in their interior. The content of physisorbed water in the samples prepared at 170 °C is always by more then one percent lower than in the samples prepared at 90 °C because these nanorods no longer form secondary structures. Methanol is released from 100 to 250 °C in the form of CO_2_ (*m*/*z* 44; Figure 8b). A step at around 250 °C corresponds to the loss of water molecules from inside the 2 × 2 channels (chemisorbed water, Figure 8b,c; *m*/*z* 18). Its content varies from 0.675% (**ND-90**) to 1.35% (**Co-90**). From the mass loss of the sample during heating from 25 to 300 °C, *n* in the formula KMn_7_^4+^Mn^3+^O_16_·*n*H_2_O can be calculated. It varies from 0.277 to 0.947. Dehydration is a topotactic reaction, and the structure remained unchanged up to 400 °C. At this temperature, the mass starts to decrease slowly, but the rate of decomposition increases at 500 to 550 °C. Due to the slow rate of the decomposition reaction, onset temperatures are not determined. From Figure 8a one may observe that the thermal stability of the samples (within a separate batch) prepared at higher reaction temperatures is slightly higher. In this step, which is completed between 600 and 700 °C, α-MnO_2_ directly transforms to Mn_3_O_4_ without Mn_2_O_3_ as an intermediate phase (proved by XRD, results not shown here). This behavior has been observed also by other authors [5,9]. The mass loss in this step varies from 7.98 to 9.42%. During this step, the evolution of oxygen (*m*/*z* 32) was detected in the mass spectrometer. The fourth step of thermal decomposition, from 700 °C to 900 °C, occurs only in some cases (comparison of Figure 8b and Figure 8c; in the former, the fourth step takes place, whereas in the latter not). In this temperature range, thermal decomposition of sulfate groups occurred, leading to the evolution of SO (*m*/*z* 48) and SO_2_ (*m*/*z* 64) gasses (Figure 8b), meaning that these groups were present in the samples in different proportions. They originate from the sulfuric acid, which was part of the reaction mixture, and their contents may differ due to slightly different washing procedures at the end of the preparation route.

## Conclusion

Regardless of the reaction temperature, all prepared samples (undoped, cobalt- and chromium-doped) exhibit the cryptomelane (α-MnO_2_-type) structure. α-MnO_2_ products grow in a form of 1D crystalline nanorods with a diameter of 40 nm on average. The XANES results show that the oxidation state of manganese is 4+, while cobalt and chromium are in a 3+ oxidation state. Only in the **Co-90** sample (XANES results) is the oxidation state of both manganese and cobalt slightly lower, leading to increasing lattice parameter *a* in a tetragonal structure. With increasing reaction temperature, the crystallinity of nanorods improves while incorporation of Cr^3+^ and Co^3+^ is reduced. In all doped samples, chromium content is much lower than that of cobalt, although the initial molar concentration in the reaction mixture was the same for both ions. As was expected from a comparison of ionic radii of manganese, potassium, and both dopant ions, the EXAFS results confirm that both dopant ions replace Mn^4+^ in the MnO_6_ octahedron, rather than K^+^, which is situated within the channels of the structure. The K/Mn ratio of the samples synthesized at 170 °C is the lowest in the **Cr-170** sample. Hence, we assume that a part of the chromium ions may substitute K^+^ ions in the channels. Furthermore, the reaction temperature has a significant impact on the secondary structure. At the lowest reaction temperature (90 °C) nanorods form hollow 3D microstructures that resemble sea urchins. At a higher reaction temperature (130 °C), the secondary structure appears to be more disordered, while it is not observed at all at 170 °C. The typical diameter of sea urchin-like structures is between 2 and 5 μm.

The course of thermal decomposition of the doped samples did not differ significantly from the undoped ones, i.e., the dopant ions in our case did not preserve MnO_2_ phase at higher temperatures nor did they destabilize the structure (in this case thermal decomposition would occur at lower temperatures for the doped samples). On heating from room temperature to around 250 °C, adsorbed water is released together with methanol. Water molecules inside the channels are topotactically released at around 250 °C. At around 400 °C, slow thermal decomposition of the cryptomelane structure begins, leading to the formation of Mn_3_O_4_. In the last step, from 700 to 900 °C, decomposition of sulfate ions occurred in some of the samples only.

## Experimental

### Materials and methods

In a typical synthesis, 1.6 mmol of KMnO_4_ (Aldrich) was dissolved in 18 mL of deionized water to form a homogeneous solution to which 0.8 mL of conc. H_2_SO_4_ (Carlo Erba) was added. The prepared reaction mixture was loaded to a 23 mL Teflon insert, and the sealed autoclave was then heated in an oven at 90, 130, or 170 °C for 10 h. After cooling down to room temperature, the resulting brown-black precipitate was collected by centrifugation, then washed first with 30 mL of deionized water, then with 30 mL of methanol, and finally dried overnight at 100 °C. In the case of doping with transition metal ions (Cr^3+^ or Co^2+^), 0.12 mmol of the corresponding salt (Cr(NO_3_)_3_·9H_2_O (Fluka) or Co(NO_3_)_2_·6H_2_O (Fluka)) was dissolved in the reaction mixture before the addition of sulfuric acid.

Undoped samples were labeled **ND-90**, **ND-130**, and **ND-170,** and the doped samples were labeled **Co**-**90**, **Co**-**130**, **Co**-**170**, **Cr**-**90**, **Cr**-**130**, and **Cr**-**170**. The figure refers to the reaction temperature.

### Characterization techniques

Powder X-ray diffraction (XRD) was performed on a PANanalytical X'Per PRO Diffractometer in the 2θ range from 5 to 80° with a step of 0.034° per second, and an integration time of 300 s. Cu Kα_1_ radiation with a wavelength of 1.5406 Å was used.

The morphology and dimensions of the isolated products were investigated with field emission scanning (FE-SEM, Carl Zeiss, Supra 35LV) and transmission electron (TEM, Jeol 2100, 200 keV) microscopes. The specimens for SEM characterization were prepared by placing a small amount of a sample into an agate mortar and dispersing it with a pestle in few drops of distilled water. A droplet of the prepared dispersion was then placed on a SEM stub covered with a carbon tape. The specimens for TEM investigations were prepared by dispersing a sample in MeOH with the help of an ultrasonic bath and depositing a droplet of the dispersion on a lacey-carbon-coated copper grid.

The elemental analysis of potassium, sulfur, manganese and cobalt/chromium was performed with the FE-SEM equipped with an energy dispersive X-ray spectrometer (EDS).

Standard K-edge X-ray absorption fine structure (XAFS) of the samples were measured at the Mn edge (6539 eV) and the dopant edges (Co: 7709 eV, Cr: 5989 eV). The **Co-130** sample was measured at the XAFS beamline of the Elettra synchrotron, Trieste, Italy, in transmission mode, while the other five samples were measured at the beamline C of Hasylab at DESY, Hamburg, Germany: Mn and Co edges in transmission mode and Cr edge in transmission and fluorescence mode due to low Cr content. For this purpose, the material was mixed with boron nitride and pressed into tablets. Separate tablets of each sample were prepared for the Mn and the dopant to optimize X-ray absorption and thus the signal-to-noise ratio. Energy calibration was established by putting the sample between the first and the second ionization chamber and the corresponding metal foil between the second and the third chambers. At least two repeated spectra were taken for each sample to check stability of the material under X-ray irradiation. The IFEFFIT program package [33] was used for the XANES (X-ray absorption near edge structure) and EXAFS (extended X-ray absorption fine structure) analysis.

High-angle annular dark field scanning transmission electron microscopy (HAADF-STEM) images were acquired using a C3/C5 Nion USTEM spherical aberration-corrected microscope working at 100 keV. Electron energy loss spectra (EELS) were recorded with a modified GATAN EELS system with a back-illuminated charge coupled device camera.

Thermogravimetric measurements were performed on a Mettler Toledo TGA/DSC1 Instrument in the temperature range from 25 to 950 °C with a heating rate of 10 K·min^−1^ in an argon atmosphere. Around 10 mg of sample was put in a 150 μL alumina crucible. The flow rate of Ar was 100 mL·min^−1^. In all the measurements the baseline was subtracted.

Analysis of the released gasses was performed by coupling the TGA/DSC1 Instrument with a quadrupole mass spectrometer Thermostar (Balzers). In this case, the initial mass of the sample was around 5 mg. Evolved gases were transferred via heated quartz capillary (*T* = 190 °C) to the entrance of the mass spectrometer.
